# Mouse strain differences in SSRI sensitivity correlate with serotonin transporter binding and function

**DOI:** 10.1038/s41598-017-08953-4

**Published:** 2017-08-17

**Authors:** Zeng-liang Jin, Xiao-Fei Chen, Yu-hua Ran, Xiao-rong Li, Jie Xiong, Yuan-yuan Zheng, Na-na Gao, Yun-Feng Li

**Affiliations:** 1 Beijing Key Laboratories of Neuropsychopharmacology, Beijing Institute of Pharmacology and Toxicology, 27 Taiping Road, Haidian, Beijing 100850 China; 2 Department of Pharmacology, School of Basic Medical Sciences, Capital Medical University, Beijing, China

## Abstract

Selective serotonin reuptake inhibitors (SSRIs) bind 5-HT transporters, leading to the accumulation of 5-HT and amelioration of depression. Although different mouse strains show varying sensitivity to SSRIs in mouse models of depression, the underlying mechanism of these strain differences remains unclear. Here, the SSRI citalopram dose-dependently reduced immobility time in both the FST and TST in DBA/2J mice but not C57BL/6J mice, whereas fluoxetine showed the opposite results. Paroxetine similarly reduced immobility time in both strains. The affinity of citalopram for the 5-HT transporter was 700-fold higher in DBA/2J mice than in C57BL/6J mice, whereas the affinity of fluoxetine was 100-fold higher in C57BL/6J mice than in DBA/2J mice. Furthermore, high citalopram concentrations were required for [^3^H]5-HT uptake in C57BL/6J but not in DBA/2J mouse cortical synaptosomes, whereas fluoxetine showed the opposite results. The effects of paroxetine on 5-HT transporter binding and synaptosomal 5-HT uptake were similar in the two strains. These results suggest that immobility duration depends on 5-HT transporter binding levels, which lead to apparent strain differences in immobility time in the FST and TST. Furthermore, differences in 5-HT transporter binding may cause variations in SSRI effects on behaviors.

## Introduction

Depression is the most prevalent psychiatric disorder, ranking among the top five leading causes of disability worldwide^1, 2^. Selective serotonin (5-HT) reuptake inhibitors (SSRIs) are widely used in the treatment of depression. However, in a fraction of patients, SSRIs are ineffective or only partially effective^3, 4^. As in other areas of medicine, the ability to predict a patient’s response to SSRIs to individually tailor treatments would be advantageous^5^. Unfortunately, the underlying mechanisms of the individual variability in SSRI response are largely unknown, although pharmacogenetic studies have linked SSRI responses to polymorphisms in genes coding for various 5-HT mechanisms, particularly the promoter of the 5-HT transporter molecule^6, 7^. The 5-HT transporter (SERT) is a key mediator of 5-HT signaling and is a major target for antidepressant medications and psychostimulants. In recent years, studies of natural and engineered genetic variations in SERT have provided new opportunities for understanding the structural dimensions of drug interactions and regulation of the transporter, for exploring 5-HT contributions to antidepressant action, and for assessing the impact of SERT-mediated 5-HT contributions to neuropsychiatric disorders^8–11^.

Depression has been frequently modeled in rodents, especially in mice^12–14^, to improve current therapeutic regimens, screen for putative antidepressant activity, or explore theories related to the etiology of depression. Mouse strain differences in immobility time and responses to antidepressants in both the forced swim test (FST) and the tail suspension test (TST) exist^15–17^. Subsequent genetic differences have been demonstrated in the performance of tests examining depression-like behavior in mice. However, why mouse strain differences are observed in the performance of these behavioral tests is unclear^18, 19^.

SSRIs strongly and selectively bind with 5-HT transporters, leading to the accumulation of 5-HT and amelioration of depression^8^. Therefore, mouse strain differences in immobility time and responses to antidepressants may be related to differences in 5-HT transporter binding. However, no study has reported 5-HT transporter binding across various mouse strains. Therefore, in the present study, we examined immobility time and locomotor activity in two mouse strains, namely, C57BL/6J and DBA/2J mice, and the effects of the SSRIs fluoxetine, paroxetine, and citalopram on these mice. Furthermore, we analyzed 5-HT transporter binding and reuptake inhibition in both strains to explore their relationship with the immobility and locomotor activity effects of the three SSRIs in these two mouse strains.

## Results

### Strain differences in SSRI effects in the tail suspension test

**Figure 1:**
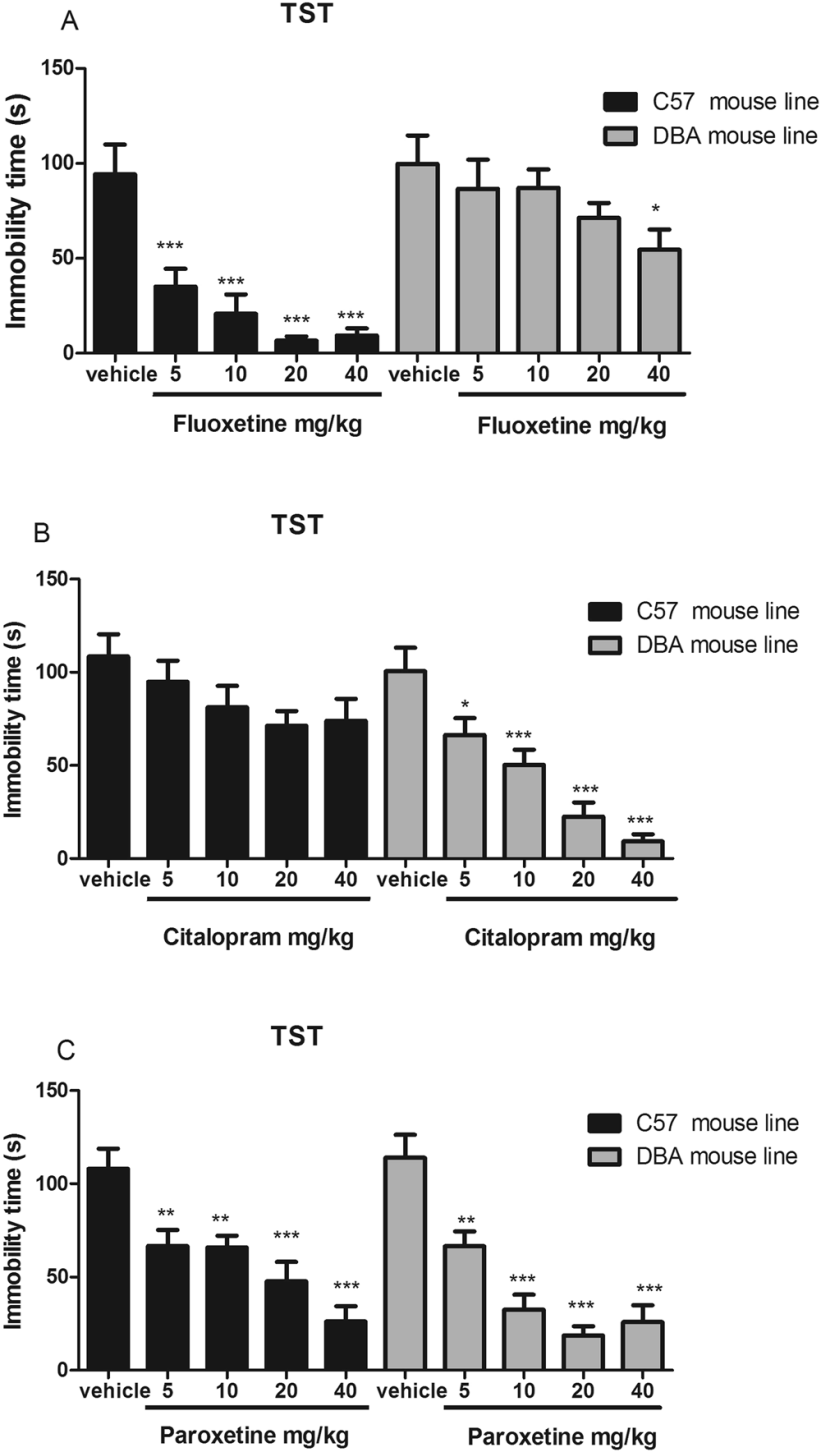
Effects of fluoxetine (**A**), citalopram (**B**) and paroxetine (**C**) on immobility time in the tail suspension test in DBA/2J and C57BL/6J mice. All tests were performed 30 min after i.p. injection of 5, 10, 20, or 40 mg/kg drug. Data are presented as the mean ± S.E.M. (n = 8–10/group). *p < 0.05, **p < 0.01, ***p < 0.001 versus vehicle.

The effects of fluoxetine, citalopram, and paroxetine in the TST differed markedly across strains (Fig. 1). Fluoxetine (5–40 mg/kg, i.p.) dose-dependently reduced immobility time in C57BL/6J mice but did not affect immobility time in DBA/2J mice, as shown in Fig. 1A. Citalopram (5–40 mg/kg, i.p.) reduced immobility time in DBA/2J mice but not in C57BL/6J mice, as shown in Fig. 1B. Paroxetine similarly reduced immobility time in both mouse strains, as shown in Fig. 1C.

### Strain differences in SSRI effects in the forced swim test

**Figure 2:**
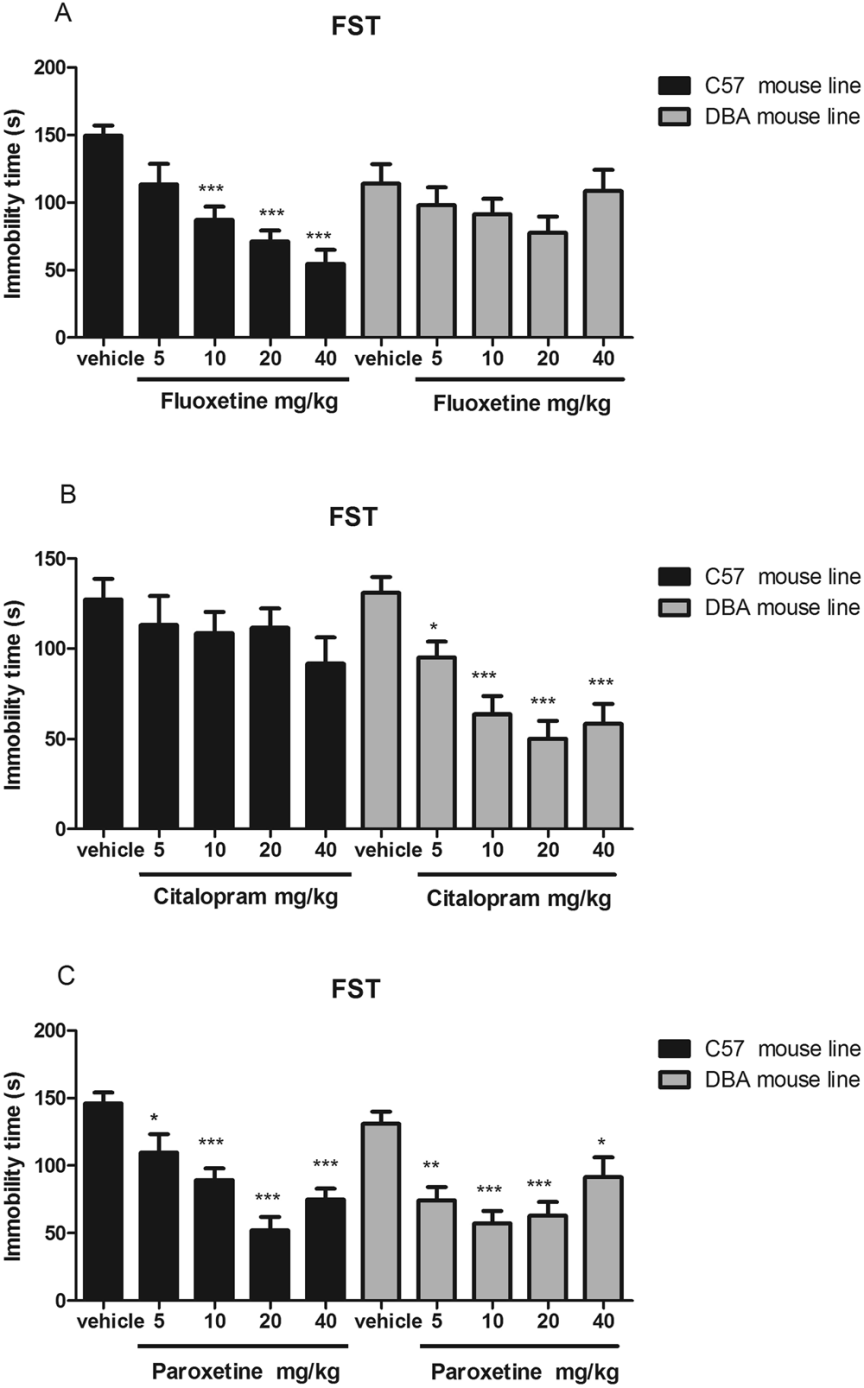
Effects of fluoxetine (**A**), citalopram (**B**) and paroxetine (**C**) on immobility time in the forced swimming test in DBA/2J and C57BL/6J mice. All tests were performed 30 min after i.p. injection of 5, 10, 20, or 40 mg/kg drug. Data are presented as the mean ± S.E.M. (n = 8–10/group). *p < 0.05, **p < 0.01, ***p < 0.001 versus vehicle.

The effects of fluoxetine, citalopram, and paroxetine in the FST differed markedly across strains (Fig. 2). Fluoxetine (10–40 mg/kg, i.p.) reduced immobility time in C57BL/6J mice but did not affect it in DBA/2J mice, as shown in Fig. 1A. Citalopram (5–40 mg/kg, i.p.) reduced immobility time in DBA/2J mice but not in C57BL/6J mice, as shown in Fig. 2B. Paroxetine similarly reduced immobility time in both mouse strains as shown in Fig. 1C.

### Fluoxetine, citalopram, and paroxetine do not affect locomotor activity in C57BL/6J or DBA/2J mice

**Figure 3:**
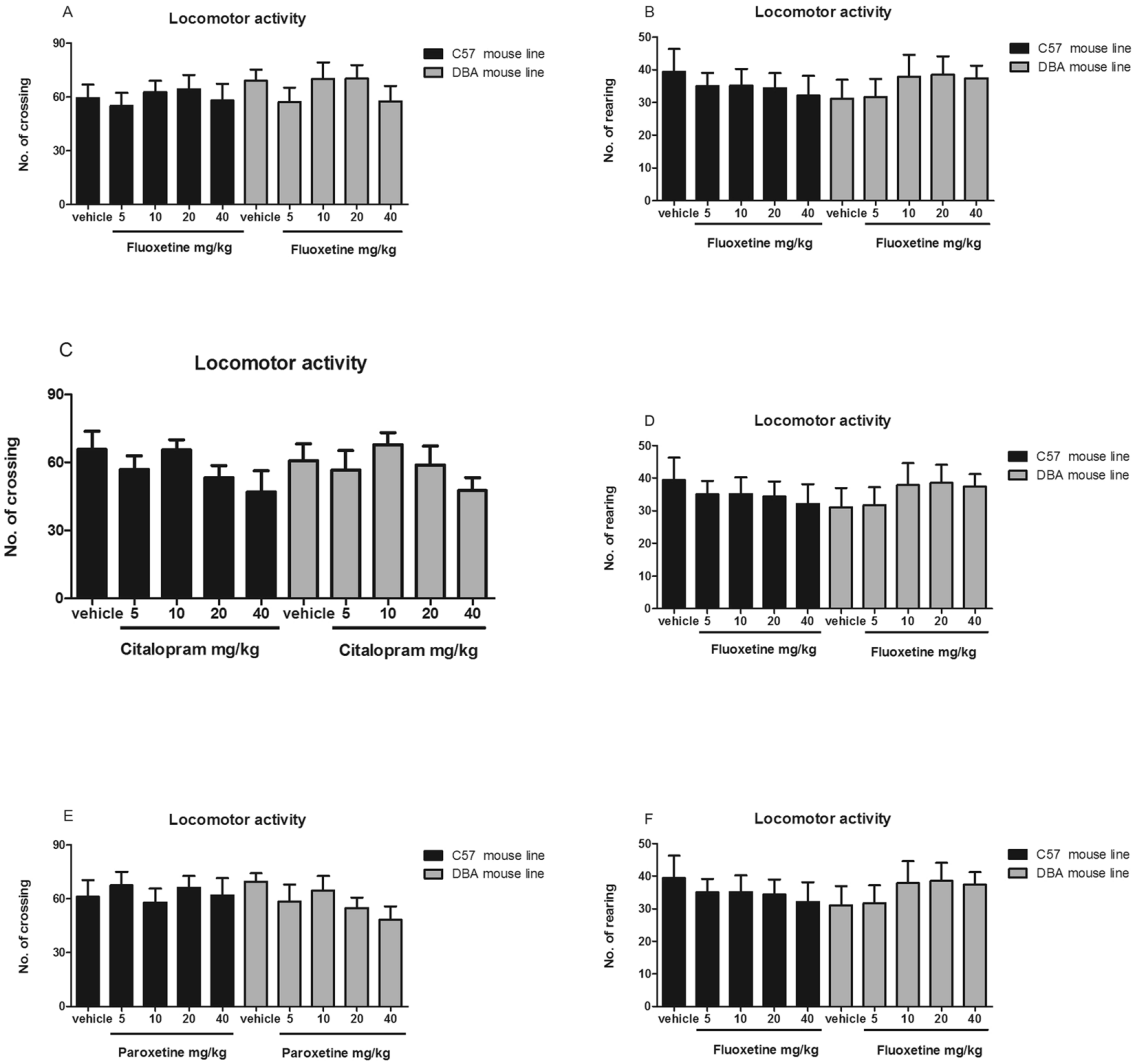
Effects of fluoxetine (**A**,**B**), citalopram (**C**,**D**) and paroxetine (**E**,**F**) on immobility time in the forced swimming test in DBA/2J and C57BL/6J mice. All tests were performed 30 min after i.p. injection of 5, 10, 20, or 40 mg/kg drug. Data are presented as the mean ± S.E.M. (n = 8–10/group). *p < 0.05, **p < 0.01, ***p < 0.001 versus vehicle.

The effects of fluoxetine, citalopram, and paroxetine on locomotor activity were examined in C57BL/6J and DBA/2J mice. No differences in locomotion were found between the mouse strains at baseline, as shown in Fig. 3. In addition, compared with administration of saline, administration of four different doses of fluoxetine, citalopram, or paroxetine failed to affect the number of crossings or rearings in each mouse strain.

### No strain differences in 5-HT transporter protein levels and kinetic constants

**Figure 4:**
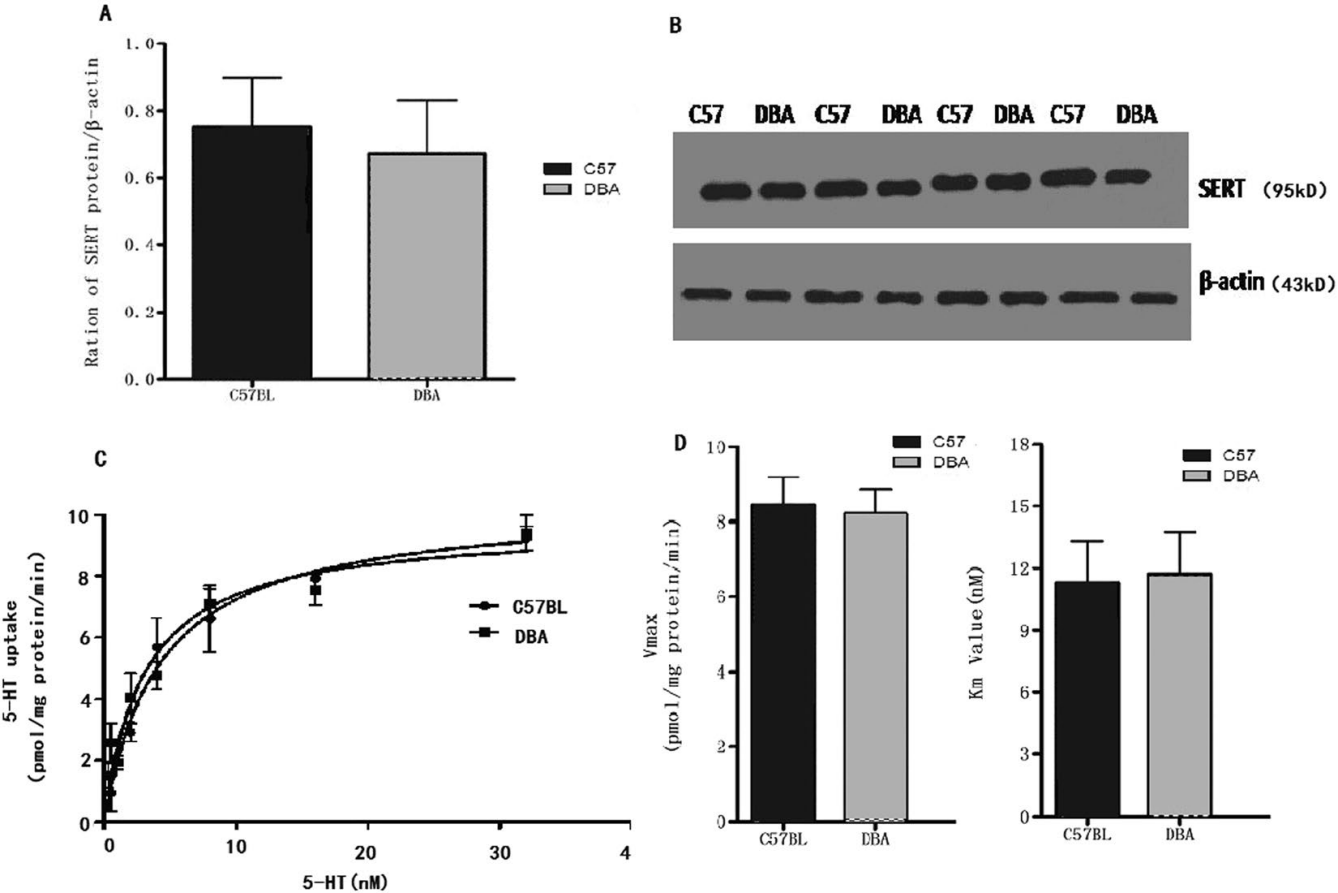
SERT protein expression (**A**) was measured by Western blotting (right) and normalized to β-actin levels (**B**); the samples were derived from the same experiment, and the gels were processed in parallel. SERT expression did not differ between C57BL/6J and DBA/2J mice (Student’s t-test, *p* > 0.05; *n* = 4 per strain). Saturation uptake kinetics in C57BL/6J and DBA/2J mice whole-brain synaptosomes (**C**,**D**). C57BL/6J and DBA/2J mice do not differ in 5-HT transport activity (C57BL/6J:V_m_ 8.23 ± 2.28 nM; K_max_ 6.54 ± 1.27 pmol/min-mg protein; DBA/2J: K_m_7.51 ± 2.67 nM; V_max_12.22 ± 3.09 pmol/min-mg protein: Student’s t-test, *p* > 0.05, *n* = 6 per strain).

To initiate our studies, we sought to confirm that SERT strain variation did not alter SERT protein expression, 5-HT recognition, or uptake activity when expressed in C57BL/6J and DBA/2J mice. Hippocampal 5-HT transporter protein levels are shown in Fig. 4A,B; no significant difference was detected in 5-HT transporter protein levels between the strains. For the synaptosomal 5-HT transporter, V_max_ and K_m_ values were determined by curve-fitting of the data to the Michaelis-Menten equation as previously described^14^. Similarly, we found no genotype effects on *ex vivo* 5-HT transport kinetics in whole-brain synaptosomes in 5-HT K_m_ or in 5-HT transport V_max_ (Fig. 4C,D).

### Mouse strain differences in SSRI-specific binding to the 5-HT transporter

**Figure 5:**
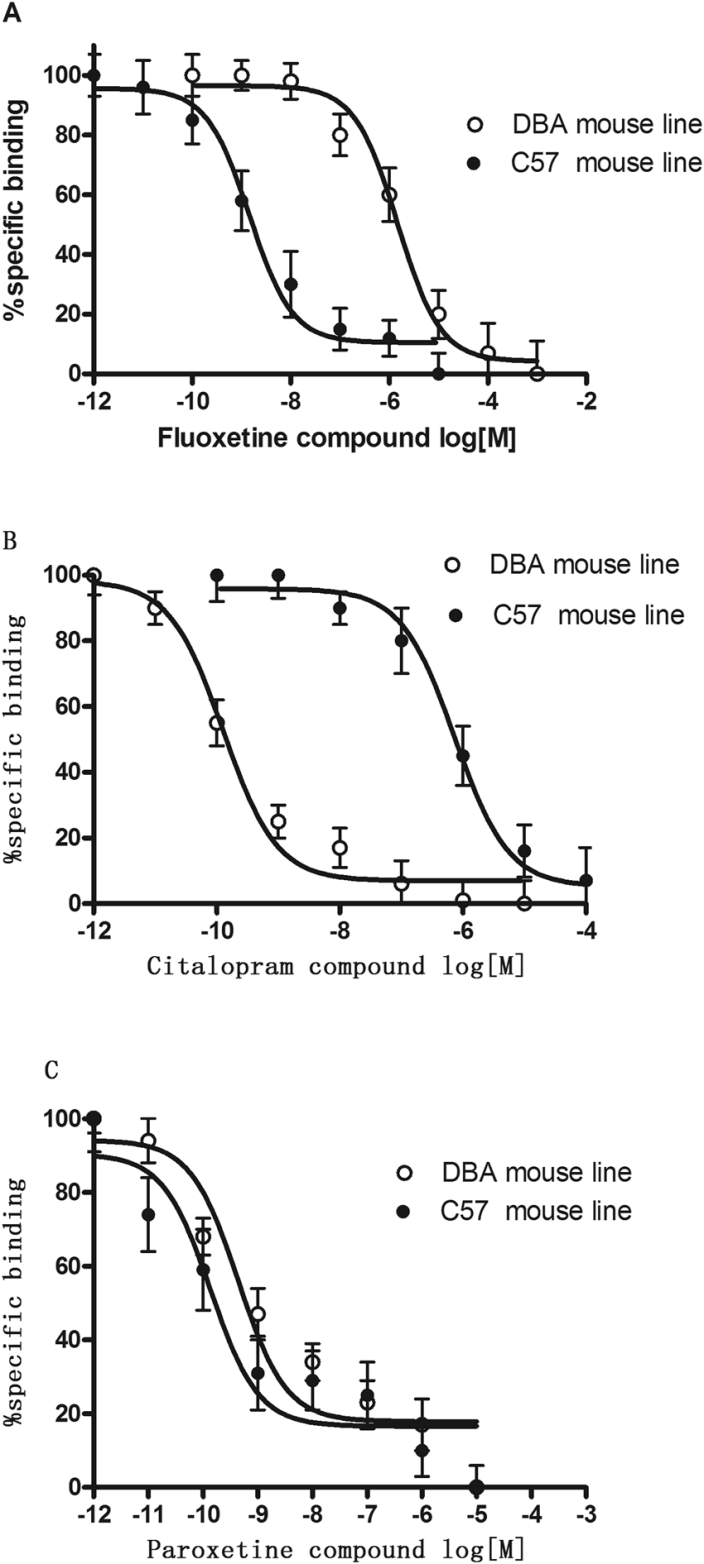
Fluoxetine (**A**), citalopram (**B**) and paroxetine (**C**) compete for 5-HT transporter binding (specific [^3^H]paroxetine binding) in the two mouse strains. The IC_50_ value was generated from each of these curves. Each data point depicted represents the mean ± S.E.M. of 3–4 mice. The IC_50_ value for fluoxetine, citalopram and paroxetine are shown in Table 1.

Radioligand binding assays were conducted to determine the affinity of the SSRIs for the 5-HT transporters in the two mouse strains. Figure 5 and Table 1 show the K_i_ values of the SSRIs for 5-HT transporter binding (specific [^3^H]paroxetine binding) in the two mouse strains. Paroxetine potently and concentration-dependently inhibited the binding of [^3^H]paroxetine to the 5-HT transporter in DBA/2J and C57BL/6J mice, with K_i_ values of 0.45 and 0.14 nM, respectively. However, fluoxetine showed low affinity for the DBA/2J mouse 5-HT transporter, with a K_i_ value of 132 nM, which was 100-fold lower than that for the C57BL/6J mouse 5-HT transporter (1.47 nM). By contrast, citalopram showed low affinity for the C57BL/6J 5-HT transporter, with a K_i_ value of 734 nM, which was 700-fold lower than that for the DBA/2J mouse 5-HT transporter (1.28 nM).

**Table 1:** The specific parameters in binding and uptake assays.

	Binding K_i_ (nM)		[^3^H]5-HT uptake IC_50_ (nM)	
	C57BL6	DBA/2J	C57BL6	DBA/2J
Fluoxetine	1.47 ± 0.25	132 ± 0.35***	2.74 ± 0.85	860 ± 24.45***
Citalopram	734 ± 28.7	1.28 ± 0.58***	214 ± 27.75	1.34 ± 0.35***
Paroxetine	0.45 ± 0.21	0.14 ± 0.05	0.85 ± 0.15	0.57 ± 0.27

Fluoxetine, citalopram and paroxetine compete for 5-HT transporter binding (specific [^3^H]paroxetine binding) and 10 nM [^3^H]5-HT reuptake into hippocampal synaptosomes in the two mouse strains. The IC_50_ value was generated from each of these curves. Each data point depicted represents the mean ± S.E.M of 3–4 mice. ***p < 0.001, Student’s *t*-test.

### Strain differences in inhibition of 5-HT uptake by SSRIs

**Figure 6:**
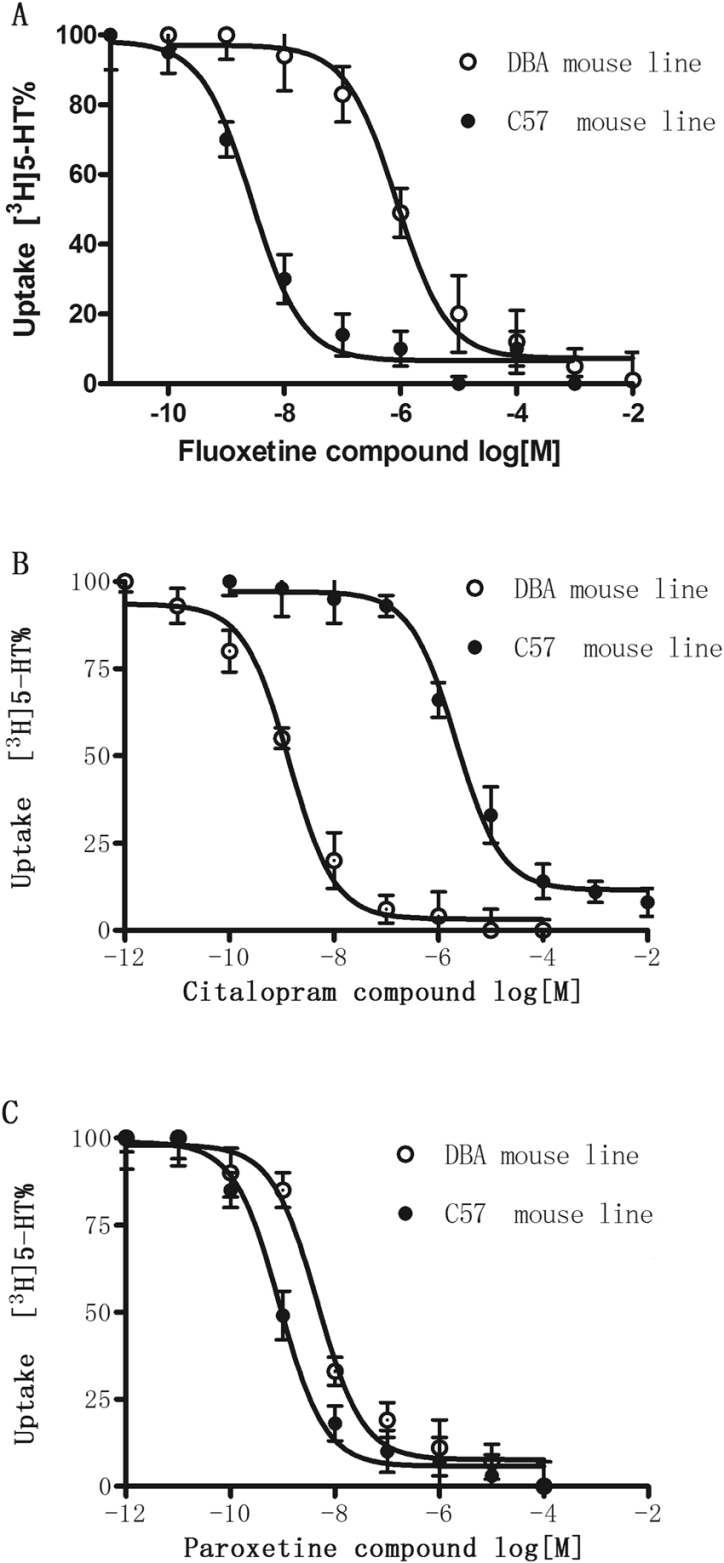
Effects of fluoxetine (**A**), citalopram (**B**) and paroxetine (**C**) on 10 nM [^3^H]5-HT reuptake into hippocampal synaptosomes from DBA/2J and C57BL/6J mice. Each value is the mean ± S.E.M. of 3–4 mice. The IC_50_ values for fluoxetine, citalopram and paroxetine are shown in Table 1.

The inhibitory effect of SSRIs on [^3^H]5-HT uptake was examined in cortical synaptosomes derived from DBA/2J and C57BL/6J mice. Consistent with its affinity binding profile at SERT, paroxetine displayed potent inhibition of [^3^H]5-HT uptake into cortical synaptosomes of DBA/2J and C57BL/6J mice, with IC_50_ values of 0.85 and 0.57 nM, respectively, as shown in Fig. 6 and Table 1. Consistent with its affinity binding profile for SERT, high concentrations of fluoxetine were required to alter [^3^H]5-HT uptake in DBA/2J mouse cortical synaptosomes, with an IC_50_ value of 860 nM, which was 300-fold higher than that for the C57BL/6J mouse (2.74 nM). In contrast to fluoxetine, high concentrations of citalopram were required to alter [^3^H]5-HT uptake in C57BL/6J mouse cortical synaptosomes, with an IC_50_ value of 214 nM, which was 200-fold higher than that for the DBA/2J mouse (1.34 nM).

## Discussion

The FST and TST evaluate the efficacy of antidepressants in rodents. Many antidepressants decrease immobility time in these two tests^13, 19, 20^. Moreover, the DBA/2J and C57BL/6J mouse strains have been widely used for the identification of novel SSRIs. The inbred and clonal nature of mouse strains commonly used by neuroscientists provides an important opportunity to limit variation in experiments and to thereby reduce the numbers of animals that must be tested to provide confidence in results. In the present study, the DBA/2J and C57BL/6J mouse strains showed significant differences in their responses to the SSRIs fluoxetine, citalopram, and paroxetine. We found strain differences in the sensitivity of C57BL/6J and DBA/2J mice to the effects of the SSRIs fluoxetine and citalopram on the immobility time in the FST and TST. The C57BL/6J mice responded to the lowest dose of fluoxetine (5 mg/kg), whereas DBA/2J mice responded to only the highest dose of fluoxetine studied (40 mg/kg). By contrast, DBA/2J mice responded to citalopram at the lowest dose (5 mg/kg), whereas C57BL/6J mice showed no response to citalopram, even at the highest dose (40 mg/kg). No strain differences were observed in the sensitivity to paroxetine in the FST and TST. Because drugs with CNS stimulant effects can decrease immobility time in the TST or FST, we also measured the effects of SSRIs on locomotor activity to eliminate any false-positive activity in the two mouse strains. If fluoxetine and citalopram differentially affect locomotor activity in the two mouse strains, in addition to their differential anti-immobility effects in the FST and TST, then the distinct effects of fluoxetine and citalopram on motor activity would be difficult to differentiate. Thus, we measured locomotor activity following SSRI treatments administered at five doses; we observed no change in the locomotor activity of mice treated with SSRIs compared with that of mice treated with saline in each strain. Therefore, locomotor activity was not related to the anti-immobility effect in the FST and TST elicited by fluoxetine and citalopram in the two mouse strains. Our findings of strain differences in the sensitivity to SSRIs are generally consistent with the results of Lucki *et al*., who showed that C57BL/6J mice do not respond to citalopram in the TST^21, 22^. However, in this study, C57BL/6J mice responded to fluoxetine and paroxetine, indicating that these SRRIs act via different mechanisms to alter immobility responses in the TST and FST. Therefore, strain differences should be considered when identifying novel SSRI differences, especially in the duration of baseline immobility and sensitivity to SSRIs.

To explore potential mechanisms underlying the strain differences observed in the present study, we determined whether immobility was correlated with SERT protein levels, 5-HT uptake, or the results of our SERT binding assays. The presynaptic 5-HT transporter is a key regulator of 5-HT signaling and is a major target for antidepressant medications and psychostimulants^23^. In recent years, natural and engineered genetic variations in the 5-HT transporter have provided new opportunities to understand the structural dimensions of drug interactions and regulation of the transporter, to explore 5-HT contributions to antidepressant action, and to assess the impact of SERT-mediated 5-HT contributions to neuropsychiatric disorders^12, 23^. The role of SERT in restricting the access of 5-HT to targets and in acquiring 5-HT for release has been well studied, but the acquisition of 5-HT for transglutaminase II-catalyzed covalent attachment to small GTP-binding proteins that can modulate the fusion of secretory granules is less understood^5, 7, 23^. SERT inhibitors, including SSRIs, are well known for their use in the treatment of anxiety disorders, depression, and obsessive-compulsive disorders. Naturally, polymorphisms in the SERT gene have been extensively studied in humans as potential risk determinants of neuropsychiatric disorders^19^. The Blakely lab used cross-species chimera and site-directed mutagenesis methods to establish that high-affinity antagonist recognition depends on a single residue near the 5-HT binding site (Ile172 in human and mouse and Met167 in fly)^7, 8, 23^. Site-directed mutagenesis studies revealed that a Met172 substitution in either human or mouse SERT reduces potency for many (but not all, e.g., paroxetine) SERT antagonists without affecting the interactions of 5-HT or other substrates^5, 7, 8^. The present results showed no changes in 5-HT reuptake in saturation uptake kinetics between strains, which is consistent with the Blakely lab studies^7^. Immobility time was not significantly correlated with the 5-HT transporter protein expression level, V_max_, or K_m_, suggesting that the membrane-bound 5-HT transporter was not associated with the strain differences in immobility. Because we found clear strain differences in response to the SSRIs, we also examined 5-HT transporter binding of the SSRIs in the brains of the two strains of mice, as SSRIs bind the 5-HT transporter, leading to elevation of monoamines and antidepressant effects^9, 15, 24^. We found that paroxetine demonstrated equivalent high affinity for SERT in the two strains of mice. Fluoxetine demonstrated high affinity for SERT in C57BL/6J mice, which was 100-fold higher than that for SERT in DBA/2J mice. By contrast, citalopram demonstrated high affinity for the DBA/2J mouse SERT, which was 700-fold higher than that for the C57BL/6J mouse SERT. The pattern of these results is similar to that of the behavioral assays. Moreover, the results of the binding assays are consistent with those from transporter functional studies, showing a similar pattern in the capacity of SSRIs to inhibit [^3^H]5-HT reuptake in synaptosomes derived from the two mouse strains. Thus, the 5-HT uptake assays demonstrated that in accordance with its affinity for SERT, paroxetine potently inhibited the uptake of [^3^H]5-HT into mouse synaptosomes derived from both C57BL/6J and DBA/2J mouse strains, with no difference between the strains. Fluoxetine also exerted potent inhibitory effects on [^3^H]5-HT uptake in the C57BL/6J mouse strain, but this effect was 300-fold greater than that in the DBA/2J mouse strain. Consistent with the results obtained in binding assays, citalopram inhibited 5-HT with 200-fold greater efficacy in the DBA/2J mouse strain than in the C57BL/6J mouse strain. Changes in the FST and TST are usually linked to SERT function/binding properties, but the circuit underlying these behaviors is unknown. These changes may be due to differences in the brain regions in which SERT activity was analyzed. Our studies were performed in the hippocampus, a discrete brain region that is clearly involved in the pathophysiology of depression. Since it is impossible to predict changes in hippocampal SERT binding based on the results of other brain regions, relevant comparisons cannot be made. Our studies are limited to data obtained from the hippocampus using Western blot analysis and synaptosomal preparations. Therefore, the contributions of different brain regions to these behaviors require further study.

In conclusion, mouse strain differences in the sensitivity to SSRI treatments were correlated with 5-HT transporter binding and reuptake in these mice, suggesting that the binding and uptake efficiency of the membrane-bound 5-HT transporter may be significant contributing factors to the marked strain differences in immobility time in the TST and FST. SERT protein mutants appear to be responsible for the mouse strain differences in altered potency to SSRIs^7, 11, 23, 25^. In the Blakely lab, site-directed mutagenesis studies revealed that a Met172 substitution in mouse SERT reduces potency for many SERT inhibitors without affecting the interactions of 5-HT or other substrates. Moreover, they developed a knock-in mouse model in which high-affinity interactions of many antidepressants with SERT were ablated via knock-in substitution without disrupting 5-HT binding or uptake. They utilized the C57BL/6J SERT mutation model to evaluate the SERT dependence of the actions of two SSRIs, fluoxetine and citalopram, in tests sensitive to acute and chronic actions of antidepressants. In the TST and FST, fluoxetine and citalopram failed to reduce immobility in SERT mutation mice. In addition, SERT mutation mice were insensitive to chronic fluoxetine and citalopram administration in the novelty induced hypophagia test and failed to exhibit enhanced proliferation or survival of hippocampal stem cells. In both acute and chronic studies, SERT mutation mice maintained sensitivity to paroxetine, an antidepressant that is unaffected by the mouse mutation^7^. Therefore, the background strain of these mice likely contributes to the acute behavioral actions of SSRIs in immobility time^7, 14, 18^. These differences may help to explain some of the discrepancies in studies that used these strains of mice to examine the role of 5-HT in mouse models of depression^26–28^. Future studies should investigate additional neural substrates and molecular mechanisms underlying strain variations in mouse models of depression to help identify genetic predispositions to this disorder in humans.

## Methods

### Drugs and reagents

Fluoxetine, paroxetine, and citalopram were purchased from Sigma and dissolved in saline. [^3^H]paroxetine and [^3^H]5-HT were purchased from PerkinElmer Life Sciences (New England Nuclear Corporation, Boston, MA).

### Animals

Male C57BL/6J and DBA/2J mice, aged 5–7 weeks, 16–20 g, were purchased from Beijing Vital River Laboratory Animal Technology Company (Beijing, China). Mice were housed in groups of five in a controlled facility with a 12-h light-dark cycle (lights on at 7:00 a.m.), room temperature of 23 ± 1 °C, and humidity of 55% ± 5%. The mice were given free access to food and water. Each mouse was used for only one experiment. All experiments were performed in accordance with relevant guidelines and regulations approved by the Experimental Animal Research Committee of Beijing Institute of Pharmacology and Toxicology.

### Forced swim test

Fluoxetine, paroxetine, and citalopram were administered to mice via intraperitoneal (i.p.) injections at doses of 5–40 mg/kg. Mice in the control group received the same volume of saline. Thirty minutes after SSRI treatment, the FST was conducted. The test was performed based on the procedure described by previous with a few modifications^16, 20^. Mice were individually placed in a plastic cylinder (height = 45 cm, diameter = 19 cm) containing water (height = 23 cm) maintained at 23 ± 1 °C for 6 min and were scored for immobility during the last 4 min. Immobility was defined as the absence of active, escape-oriented behaviors, with only small movements to keep the head above water.

### Tail suspension test

Fluoxetine, paroxetine, and citalopram were administered to mice via intraperitoneal (i.p.) injections at doses of 5–40 mg/kg. Mice in the control group received the same volume of saline. Thirty minutes after SSRI treatment, the TST was conducted as described by previous^16, 20^. Briefly, mice were individually suspended from the edge of a shelf, 60 cm above the ground, by adhesive tape placed over the tail at 1 cm from the tip. Mice were suspended for 6 min and were scored for immobility during the last 4 min. Immobility was defined as motionless without any agitation.

### Locomotor activity measurement

Thirty minutes after SSRI treatment, mice were placed in the corner of a 36 × 29 × 23 cm plastic box (the base was divided into equal sections) for a 5-min acclimation. The numbers of crossings (crossing outside the section with four paws) and rearings (raising the forepaws) were recorded for the subsequent 5 min.

### Western blot analysis of 5-HT transporter protein

C57BL/6J and DBA/2J mice were used in Western blot experiments. Each dissected hippocampal subregion was homogenized in ice-cold lysis buffer (0.1% SDS, 1% Triton X-100, 10 mM HEPES, 5 mM NaF, and 0.25 M sucrose, pH 7.4) to prepare the cell lysate. Protein concentration was measured using the Bradford method (Bradford, 1976), with bovine albumin as the standard. Each sample containing 100 μg of protein was loaded into a 12% polyacrylamide gel, separated by sodium dodecyl sulfate-polyacrylamide gel electrophoresis, and then transferred to a polyvinylidene difluoride membrane (Millipore, USA) overnight. The membrane was blocked with 5% milk in Tris-buffered saline with Tween 20 (TBS-T) for 2 h at room temperature. A goat anti-SERT polyclonal antibody (1:300, Santa Cruz) was incubated with the membrane for 4 h at room temperature. The membrane was washed once for 30 min and twice for 20 min with TBS-T and incubated with a horseradish peroxidase-conjugated anti-goat IgG secondary antibody (1:500, Santa Cruz) for 2 h at room temperature. After the membrane was washed again, specific immunoreactive staining was visualized by enhanced chemiluminescence detection reagents (Sigma, USA) and captured using a Tanon 4200 Chemiluminescent Imaging System (Tanon Science, China). The housekeeping protein β-actin was detected with a polyclonal rabbit antibody (1:1,000, Santa Cruz) and a secondary anti-rabbit antibody (1:1,000, Santa Cruz). SERT was revealed as a band of 70 KDa and β-actin as a band of 42 KDa. Band density was analyzed with GIS image analysis software (Tanon Science, China). To eliminate possible variations in the efficiency of protein extractions and sample loading, β-actin was used as an internal control. The expression level of SERT was normalized to the corresponding β-actin level in each sample.

### 5-HT transporter radioligand binding assay

The 5-HT transporter binding assay was conducted as previously described using [^3^H]paroxetine in two mouse strains^16^. Briefly, mice of each strain were decapitated, and their brains were removed and stored at −80 °C until analysis. Brain tissue was homogenized in 19 volumes of 50 mM Tris-HCl buffer (pH 7.4). Competitive binding assays were performed as previously described^16^ using [^3^H] paroxetine (1.2 nM) as the radioligand and 10 mM fluoxetine to define non-specific binding. Binding assays were performed in duplicate in three independent experiments. The apparent dissociation constant (K_d_) and maximal number of binding sites (B_max_) for [^3^H]paroxetine were estimated by Rosenthal analysis of the saturation data (Rosenthal, 1967).

### Synaptosomal 5-HT uptake assay

[^3^H]5-HT uptake assays were performed using crude synaptosomes prepared from the brain tissue of the two mouse strains, as described in previous reports^7, 16^. Crude synaptosomes were incubated in Krebs bicarbonate solution containing drug solution and [^3^H]5-HT (20 nM) for 10 min at 37 °C. Non-specific uptake was determined using 10 mM fluoxetine or desipramine. The uptake assays were performed in duplicate in three independent experiments. For saturation uptake assays, synaptosomes were prepared using the whole brain. Synaptosomes were incubated at 37 °C for 5 min with serial dilutions of 5-HT stock containing 10% [^3^H]5-HT. At each concentration of 5-HT, parallel samples were incubated in the presence of 1 μM paroxetine, defining non-specific uptake, which was subtracted from the total counts to yield specific uptake. Synaptosomes were assessed with protein concentration for the normalization of 5-HT levels across experiments (Pierce BCA; ThermoFisher).

### Statistical analysis

The behavioral study results are expressed as the mean ± standard error of the mean (S.E.M.) of 8–10 mice. The transporter binding study results are shown as the mean ± S.E.M. of 3–4 experiments. The dose-related effects of fluoxetine, citalopram, and paroxetine on immobility and locomotor activity were evaluated using one-way analysis of variance (ANOVA) followed by Dunnett’s test. Unless otherwise specified, statistical analyses were performed using GraphPad Prism (GraphPad Prism 5.0, version 2.0; GraphPad Software Inc., San Diego, CA). The transporter binding and monoamine uptake data were analyzed using one-site nonlinear regression of the concentration-effect curve. The K_i_ values were calculated using the Cheng-Prusoff equation as follows: K_i_ = IC_50_/[(L/K_d_) + 1] (Cheng and Prusoff, 1973). The IC_50_ values in the 5-HT uptake tests were calculated using the method described by Bliss (1967)^29, 30^.
